# Dyspnea is associated with poor physical performance among community-dwelling older adults: a population-based cross-sectional study

**DOI:** 10.1590/1516-3180.2019.0428.R1.09122019

**Published:** 2020-04-09

**Authors:** Caroline de Fátima Ribeiro Silva, Maycon Sousa Pegorari, Areolino Pena Matos, Daniela Gonçalves Ohara

**Affiliations:** I PT. Physiotherapist and Student, Postgraduate Program on Health Science, Universidade Federal do Amapá (UNIFAP), Macapá (AP), Brazil.; II PhD. Physiotherapist and Adjunct Professor, Physiotherapy Course, Universidade Federal do Amapá (UNIFAP), Macapá (AP), Brazil.; III PhD. Physiotherapist and Adjunct Professor, Physiotherapy Course, Universidade Federal do Amapá (UNIFAP), Macapá (AP), Brazil.; IV PhD. Physiotherapist and Adjunct Professor, Physiotherapy Course, Universidade Federal do Amapá (UNIFAP), Macapá (AP), Brazil.

**Keywords:** Dyspnea, Physical functional performance, Frail elderly, Physical performance, Older adults, Stress test, Short physical performance battery

## Abstract

**BACKGROUND:**

Dyspnea and poorer physical performance are conditions that may be related and be present among the elderly. However, few studies have evaluated associations between these variables.

**OBJECTIVE:**

To determine whether there is an association between dyspnea and physical performance among community-dwelling older adults of both sexes (age 60 years and over).

**DESIGN AND SETTING:**

Cross-sectional study conducted in the city of Macapá, state of Amapá, Brazil.

**METHODS:**

Socioeconomic and health data were collected using a structured form. Frailty syndrome was assessed based on the frailty phenotype proposed by Fried et al. Dyspnea was measured using the modified Medical Research Council (mMRC) scale and physical performance was measured using the Short Physical Performance Battery (SPPB). Data were analyzed using a linear regression model.

**RESULTS:**

A total of 411 subjects (70.15 ± 7.25 years) were evaluated, most of them females (66.4%). It was observed from the mMRC scale that 30.9% (n = 127) of the subjects had some dyspnea symptoms: grade 1 was most frequent. The physical performance score from the SPPB was 9.22 ± 2.01. Higher dyspnea scores were associated with poor physical performance, both in the crude analysis (β = -0.233; P = 0.028) and after adjustment for frailty condition (β = -0.148; P = 0.002) and for the socioeconomic and health variables (age, sex, number of diseases, smoking habit and frailty status) (β = -0.111; P = 0.025).

**CONCLUSION:**

Higher dyspnea score was independently associated with poor physical performance among community-dwelling older adults.

## INTRODUCTION

Dyspnea is a highly prevalent and common symptom among older adults.^1^ Presence of this symptom may be disabling, since it is associated with limited mobility, functional decline and frailty in this population.^2^

According to Mahler,^3^ approximately 30% of the population of adults older than 65 years report dyspnea during daily activities such as walks and/or uphill climbing. Among the factors that favor the onset of this symptom, reduced exercise tolerance and a low level of physical activity seem to contribute to a decline in physical performance during activities of daily life.^2^^,^^4^

Vaz Fragoso et al.^4^ and Larsson et al.^5^ reported that poor physical performance assessed on the basis of lower-limb performance in physical tests such as sitting and rising from a chair is associated with an increase in dyspnea among both robust older adults and older adults with frailty and/or chronic obstructive pulmonary disease (COPD).

However, it should be pointed out that few studies evaluating dyspnea and physical performance are available. There is some mention of the health-impairing consequences of dyspnea or poor physical performance in the literature, but only under specific conditions involving populations of older subjects with COPD, postoperative situations, frailty and depressive symptoms.^5^^,^^6^^,^^7^

Since dyspnea is a nonspecific symptom associated with adverse outcomes such as exercise intolerance, physical disability and increased mortality among older adults, investigation of dyspnea, allied with evaluation of physical performance, is important. It needs to be borne in mind that a large proportion of older adults who report dyspnea do not have any previously installed cardiopulmonary impairment.^4^

Thus, dyspnea can become a starting point for identifying possible changes that affect older adults under both specific and nonspecific conditions. These conditions include frailty and situations of age-related increases in multimorbidity. In addition, evaluation of dyspnea is done through a simple measurement that is easy to perform, such that dyspnea is easily identifiable.^8^

## OBJECTIVE

The objective of the present study was to determine whether there is any association between dyspnea and physical performance among community-dwelling older adults.

## METHODS

### Design and sample

This was a cross-sectional study conducted among older adults living in the urban area of Macapá, a city in the Amazon region that is the capital of the state of Amapá, in northern Brazil. Information about the characteristics of the population and the sample size calculation is available in a previous study by Ohara et al.^9^

### Inclusion and exclusion criteria

Older adults aged 60 years or over who were living in the urban area of Macapá, and who were able to walk unaided or with a gait-aiding device, were included in the study. Subjects who were not located after three visits and/or subjects who were institutionalized or hospitalized at the time of the interview, or who presented neurological and/or orthopedic conditions that would prevent evaluation, were excluded. Also excluded were subjects with cognitive decline that would prevent them from answering the questions of the interview, as determined using the Mini-Mental State Examination (MMSE), in its version translated and validated for Brazilian Portuguese,^10^ which considers cutoff points according to educational level.

The study was approved (protocol no. 1.738.671; dated September 21, 2016) by the local human-research ethics committee. The older adults were recruited and assessed at their respective homes in the year 2017, and interviews were conducted face-to-face by properly trained undergraduate students and monitored by field supervisors (researcher teachers). A total of 443 older adults were recruited and assessed: 27 of these were excluded because they showed cognitive decline and 5 were excluded for other reasons such as incomplete data. After considering the eligibility and loss criteria, the present study was conducted among 411 community-dwelling older adults.

### Instruments for data collection

#### Modified Medical Research Council (mMRC) scale (independent variable)

The sensation of dyspnea was assessed using the modified Medical Research Council (mMRC) scale, which involves a score from 0 to 4. The higher the score is, the worse the sensation of dyspnea is.^11^ The older adults of this study graded their onset of dyspnea according to their physical activity, according to the following items: grade 0 (shortness of breath only during intense exercises); grade 1 (shortness of breath when walking fast or climbing uphill); grade 2 (walking more slowly due to shortness of breath or needing to stop during exercise); grade 3 (needing to stop in order to breathe after a 100 m walk); and grade 4 (shortness of breath that prevented the subject from leaving home or that occurred when changing clothes).^11^^,^^12^

#### Short Physical Performance Battery (SPPB) (dependent variable)

Physical performance was assessed using the translated version of the Short Physical Performance Battery (SPPB), which has been adapted to Brazilian realities.^13^ This consists of the following components: static standing balance, gait speed at habitual pace and five-time sit-to-stand test without the help of the upper limbs. The total SPPB score is calculated as the sum of the scores for each test and can range from 0 to 12 points. A 0 to 3-point score for the SPPB indicates disability or very poor performance; a score of 4 to 6 indicates poor performance; a score of 7 to 9 indicates moderate performance; and a score of 10 to 12 indicates good performance.^14^^,^^15^

#### Adjustment variables

Socioeconomic and health variables were assessed using a structured questionnaire. Information regarding sex, age and physical health variables such as number of diseases and smoking habit was obtained. Frailty syndrome was determined as described in previous studies^16^^,^^17^ and was based on the frailty phenotype proposed by Fried et al.,^18^ i.e. as follows:

1. Unintentional weight loss measured by means of the following question: “over the last year, did you lose more than 4.5 kg unintentionally (i.e. without diet or exercise)?”;
2. Reduced muscle strength, as assessed from handgrip strength using a manual hydraulic dynamometer (Model SH5001, SAEHAN, São Paulo, Brazil) and adopting the cutoff points proposed by Fried et al.;^18^
3. Self-reported exhaustion and/or fatigue, as measured by means of two questions from the Brazilian version of the Center for Epidemiological Studies Depression Scale (CES-D), i.e. item 7 (“Did you feel that you had to make an effort to perform your habitual tasks”) and item 20 (“Were you unable to perform your activities?”). The older subjects who obtained a score of 2 or 3 in replying to either question fulfilled the criteria of frailty for this item;
4. Slow gait speed, obtained from the gait time (in seconds) that was needed to cover a distance of 4.6 meters, using the cutoff points proposed by Fried et al.;^18^
5. Low level of physical activity, as determined using the long version of the International Physical Activity Questionnaire (IPAQ).^19^ Subjects who spent 150 minutes or more per week doing physical activity were considered to be sufficiently active, and those who spent 0 to 149 minutes per week doing physical activity were considered to be inactive.

Older subjects with three or more of these items were classified as frail and those with one or two items were classified as pre-frail, while those for whom all the tests were negative were considered to be robust or non-frail.^18^

### Statistical analysis

Descriptive statistical analysis was carried out using means, standard deviations, absolute numbers and percentages. For comparisons among groups, the chi-square test and Student’s t test were used.

Inferential analysis was performed in order to determine associations between dyspnea and physical performance. For this, crude analyses and analyses adjusted through a linear regression model were carried out, taking a 95% confidence interval (CI) and a 5% significance level (P < 0.05). The variables considered for adjustment were age, sex, number of diseases, smoking habit and frailty status. The techniques of residual analysis (normality, linearity and homoscedasticity) and multicollinearity were used to investigate the adequacy of the linear regression model and to detect correlations between its variables and its confidence level.

 The data were analyzed using the Statistical Package for the Social Sciences (SPSS), version 21.0.

## RESULTS

Table 1 shows the characteristics of the older adults of this study according to occurrences of dyspnea. A total of 411 older adults were evaluated. Most of them were women (66.4%), with a mean age of 70.15 ± 7.25 years. Among these 411 older adults, 28.7% (n = 118) were not frail, 58.4% (n = 240) were prefrail and 12.9% (n = 53) were frail, with a score of 9.22 ± 2.01 for physical performance. Most of the older adults who reported having dyspnea symptoms according to the mMRC scale were female, had a higher mean number of diseases, showed poorer physical performance and were frail.

**Table 1.** Characteristics of the elderly participants according to occurrences of dyspnea. Macapá (AP), Brazil, 2017 (n = 411)

Variables	Dyspnea (mMRC)		P	Total sample (n = 411)
	Yes 127 (30.9%)	No 284 (69.1%)		
Age (years)	70.12 ± 7.43	70.15 ± 7.18	0.970	70.15 ± 7.25
Sex				
Male	32 (25.2%)	106 (37.3%)	0.016	138 (33.6%)
Female	95 (74.8%)	178 (62.7%)		273 (66.4%)
Number of diseases	6.78 ± 3.01	4.82 ± 2.65	< 0.001	5.43 ± 2.90
Number of medications	1.84 ± 1.70	1.52 ± 1.75	0.083	1.62 ± 1.74
Smoking habit				
Yes	11 (8.7%)	28 (9.9%)	0.702	39 (9.5%)
No	116 (91.3%)	256 (90.1%)		372 (90.5%)
SPPB (score)	8.78 ± 1.99	9.41 ± 1.97	0.003	9.22 ± 2.01
Frailty status				
Non-frail	29 (22.8%)	89 (31.3%)	< 0.001	118 (28.7%)
Prefrail	68 (53.5%)	127 (60.6%)		240 (58.4%)
Frail	30 (23.6%)	23 (8.1%)		53 (12.9%)

Data are reported as n = number of subjects; mean ± standard deviation; % = percentage; SPPB = Short Physical Performance Battery; mMRC = modified Medical Research Council scale; χ^2^ test; t-test; P < 0.05.

Table 2 shows the characteristics of these older adults according to their dyspnea symptoms (from the mMRC scale). It was observed that 30.9% (n = 127) of the subjects had some dyspnea symptoms according to the mMRC, and the most frequently mentioned level was grade 1.

**Table 2.** Characteristics of the older adults according to dyspnea symptoms (from the modified Medical Research Council scale). Macapá (AP), Brazil, 2017 (n = 411)

Dyspnea (mMRC)	n (%)
0	284 (69.1)
1	93 (22.6)
2	10 (2.4)
3	19 (4.6)
4	5 (1.2)

Data are reported as n = number of subjects; % = percentage; mMRC = modified Medical Research Council scale.

Table 3 shows the association of dyspnea (mMRC) with physical performance (SPPB) among these older adults. A higher dyspnea score was associated with poor physical performance in both the crude analysis and the analysis adjusted for frailty condition and for the socioeconomic and health variables of age, sex, number of diseases, smoking habit and frailty status.

**Table 3.** Association of dyspnea (from the modified Medical Research Council scale) with physical performance (from the Short Physical Performance Battery) among older adults. Macapá (AP), Brazil, 2017 (n = 411)

Variables	SPPB							
	B	SD	β	T	P	95% CI		R^2^
						Lower limit	Upper limit	
Dyspnea (mMRC)								
Model 1	-0.549	0.113	-0.233	-4,848	< 0.001	-0.771	-0.326	0.054
Model 2	-0.348	0.109	-0.148	-3,180	0.002	-0.562	-0.133	0.172
Model 3	-0.261	0.107	-0.111	-2,436	0.015	-0.472	-0.050	0.302

SPPB = Short Physical Performance Battery; B = non-standardized coefficients; SD = standard deviation; β = standardized coefficients; T = t test; 95% CI = 95% confidence interval; R^2^ = coefficient of determination; mMRC = modified Medical Research Council scale; Model 1 = unadjusted analysis; Model 2 = analysis adjusted for frailty condition; Model 3 = analysis adjusted for socioeconomic and health variables (age, sex, number of diseases and smoking habit) and frailty status; P < 0.05.

## DISCUSSION

The present study showed that dyspnea was associated with poor physical performance among these community-dwelling older adults, even after adjustment for socioeconomic and health conditions and frailty status.

In a systematic review, Van Mourik et al.^1^ identified combined dyspnea prevalences of 36% for scores on the Medical Research Council (MRC) scale ≥ 2; 16% for MRC ≥ 3; and 4% for MRC ≥ 4. The present results revealed that 30.9% (n = 127) of the older adults evaluated reported some dyspnea symptoms according to the mMRC scale, with grade 1 being most frequently mentioned. This indicated that dyspnea was present when the subject was walking fast on a flat terrain or climbing uphill.

In a study on 4,413 subjects older than 65 years conducted by Miner et al.,^20^ dyspnea was reported by 17.5% of the participants, irrespective of whether they were in robust health or had cardiorespiratory diseases. Among the healthy older subjects assessed by those authors, presence of dyspnea seemed to be strongly associated with factors such as depression, obesity and poor physical performance in the sit-and-rise test (a test that evaluates lower limb performance). Those findings are in agreement with the results from the present study.

In our evaluation of physical performance, which was assessed using the SPPB, we obtained a score of 9.22 ± 2.01, thus indicating moderate physical performance in the study sample. This finding has clinical implications, since the physical performance evaluated by the SPPB can predict the risk of falls, functional limitations, hospitalization and death among older individuals.^21^^,^^22^

In a systematic review, Pavasini et al.^22^ observed that SPPB scores of 0-3, 4-6 and 7-9 were associated with progressive increases in the risk of all-cause mortality, regardless of the analysis using adjustment variables, whereas this was not observed for scores of 10-12. In another study, poor SPPB performance with scores of 0 to 4 was also correlated with mortality, hospitalization and functional decline.^23^

In the present study, even after adjustment for frailty and for other possible confounding factors (socioeconomic and health variables and frailty status), dyspnea continued to be associated with physical performance. This suggests that the higher the grade of dyspnea reported by the subject is, the poorer the physical performance also is.

Vaz Fragoso et al.^4^ assessed the association between performance in the sit-and-rise test and the presence of moderate to severe dyspnea among older individuals, and also inserted frailty as a covariable in the analyses. Among the results obtained, poor physical performance in the sit-and-rise test increased the likelihood of occurrence of moderate to severe dyspnea by 85% among older individuals when they exerted effort. This result suggests that an association between lower-limb function and the sensation of dyspnea was present even after adjustment for frailty condition.

Similarly, Larsson et al.^5^ detected a significant correlation between better physical performance in the sit-and rise test (assessed using the SPPB) and lower dyspnea scores (from the mMRC) among patients of average age with chronic obstructive pulmonary disease (COPD) aged on average 69 ± 6 years. Although the health condition of the population evaluated in their study differed from that of the present sample, their finding is supported by our results. This shows that the greater the sensation of dyspnea reported was, the poorer the physical performance also was, even among older subjects who did not have any respiratory diseases (such as COPD). This finding need to be considered with attention.

We believe that the possible reason for the emergence of the inverse association between dyspnea and poor physical performance in our study related to the ventilatory response of older adults to exercise. Higher levels of ventilation are required in situations of physical exertion, which exposes these individuals to ventilatory stress, as also does the slowing down of respiratory center neuromodulation that occurs during aging.^3^ This ventilatory stress gives rise to increased dyspnea upon exertion, which may lead older adults to restrict and/or impair their performance in certain daily activities.

Among the factors that may be related to poor physical performance and dyspnea, correlations between sedentarism and worsening of dyspnea and between frailty and declining muscle function have been highlighted in the recent literature. These correlations have been based on the assumptions that sedentary older individuals have higher dyspnea scores,^6^ they present a lack of physical conditioning that impairs their performance in activities requiring effort; and they may have aggravating factors such as poor life habits and associated comorbidities, along with their advanced age per se.

Vaz Fragoso et al.^24^ demonstrated an association between sedentarism, dyspnea and poor performance in the gait speed test, in which sedentary older individuals showed impaired ventilatory capacity and/or dyspnea. These variables are associated with physical inactivity and immobility. The same group^25^ also reported that, among older individuals with limited mobility, low levels of physical activity were associated with higher likelihood of hospitalization.

In addition, in a study on 565 community-dwelling older adults, Hegendörfer et al.^2^ reported that dyspnea was an independent predictor of limitations to cardiorespiratory and physical performance, and that higher dyspnea scores increased the likelihood of mortality, hospitalization and disability.

Thus, both poor physical performance and dyspnea symptoms are factors that influence the health of the older population. Their presence may result in negative outcomes such as disability, hospitalization and even death, as mentioned earlier. Hence, studies investigating these conditions are of fundamental importance for enabling better planning of healthcare for the older population.

Some limitations of the present study should be considered. The evaluations were made in the homes of these older individuals according to their availability, in a place that would be more comfortable for them. Therefore, the times and places for data collection were not controlled. Most of the participants were women; however, we believe that this limitation was minimized due to the adjustment for sex that was incorporated in the analysis. Because of the cross-sectional design of the study, it was not possible to obtain temporality information, as is done in prospective studies. Further investigations are needed in order to obtain more precise results of greater precision regarding the association of these variables, which are still scarce in the literature.

## CONCLUSION

Higher dyspnea scores were associated with poor physical performance, both in the crude analysis and in the analyses adjusted for frailty condition and for socioeconomic and health conditions. The results suggest that the higher the degree of dyspnea reported is, the poorer the physical performance among older adults will also be.
